# Associations between GLUT expression and SUV values derived from FDG-PET in different tumors—A systematic review and meta analysis

**DOI:** 10.1371/journal.pone.0217781

**Published:** 2019-06-17

**Authors:** Hans-Jonas Meyer, Andreas Wienke, Alexey Surov

**Affiliations:** 1 Department of Diagnostic and Interventional Radiology, University of Leipzig, Leipzig, Germany; 2 Institute of Medical Epidemiology, Biostatistics, and Informatics, Martin-Luther-University Halle-Wittenberg, Halle (Saale), Germany; University of Nebraska Medical Center, UNITED STATES

## Abstract

**Purpose:**

Fluorodeoxyglucose-Positron-emission tomography (FDG-PET), quantified by standardized uptake values (SUV), is one of the most used functional imaging modality in clinical routine. It is widely acknowledged to be strongly associated with Glucose-transporter family (GLUT)-expression in tumors, which mediates the glucose uptake into cells. The present systematic review sought to elucidate the association between GLUT 1 and 3 expression with SUV values in various tumors.

**Methods:**

MEDLINE library was screened for associations between FDG-PET parameters and GLUT correlation cancer up to October 2018.

**Results:**

There were 53 studies comprising 2291 patients involving GLUT 1 expression and 11 studies comprising 405 patients of GLUT 3 expression. The pooled correlation coefficient for GLUT 1 was r = 0.46 (95% CI 0.40–0.52), for GLUT 3 was r = 0.35 (95%CI 0.24–0.46). Thereafter, subgroup analyses were performed. The highest correlation coefficient for GLUT 1 was found in pancreatic cancer r = 0.60 (95%CI 0.46–0.75), the lowest was identified in colorectal cancer with r = 0.21 (95% CI -0.57–0.09).

**Conclusion:**

An overall only moderate association was found between GLUT 1 expression and SUV values derived from FDG-PET. The correlation coefficient with GLUT 3 was weaker. Presumably, the underlying mechanisms of glucose hypermetabolism in tumors are more complex and not solely depended on the GLUT expression.

## Introduction

Fluorodeoxyglucose -Positron-emission tomography (FDG-PET) is one of the most used functional imaging modality in clinical practice. The value of this imaging technique is based upon the display of glucose metabolism in vivo [1, 2]. This benefit has been extensively researched, especially in the field of oncologic imaging. The FDG uptake is routinely quantified by standardized uptake values (SUV), which is a robust and reliable imaging biomarker [1, 2].

Malignant tumors tend to show an altered, elevated glucose metabolism based upon aerobic glycolysis compared to normal tissue, which is called Warburg effect [3, 4].

Because of this fact, FDG-PET can be used in clinical routine to aid in discrimination between benign and malignant lesions [5–7], might predict treatment response [8–10] and might also be able to reflect histopathology parameters of tumors [11, 12].

The accumulation of the tracer FDG is acknowledged to be mainly mediated by the Glucose-transporter family (GLUT) [13, 14]. These proteins are located within the cell membranes and regulate the uptake of glucose into cells. According to the literature, especially the subtypes GLUT 1 and GLUT 3 are the most important proteins for the FDG-uptake and are overexpressed in tumors [14].

In brief, a tumor cell needs more glucose for proliferation and because of the ineffective aerobic glycolysis than a physiological cell. Thus, tumors might also express more GLUT proteins than physiological tissues to accumulate more glucose.

Moreover, it was identified that an increased glucose uptake is associated with chemotherapy resistance of gemcitabine in pancreatic cancer cells [15]. A key regulator is hypoxia-inducible factor 1-alpha, which mediates the metabolic pathways, including GLUT expression [15]. In another study on pancreatic cancers it was identified that GLUT 1 expression was abundantly higher in tumors and it was even the highest expressed protein of metabolic genes [16]. These findings suggest that metabolic protein expression is associated with tumor aggressiveness and treatment response.

This association between SUV and GLUT has been extensively investigated, both in experimental animal studies [17, 18] and as well as in clinical studies using immunohistochemical stainings of tumor specimens [14]. In most studies, GLUT 1 was investigated. Previously, some studies identified a strong positive correlation between GLUT expression and SUV values derived from FDG-PET, as it is hypothetically expected [19, 20]. However, there are also studies, which could not show any significant associations between SUV and GLUT [21]. The exact reason for this discrepancy is not known. Presumably, in some tumors the FDG-PET uptake may be predominantly influenced by GLUT expression. In other malignancies, however, other cellular pathways, such as the expression of hexokinase II, may be more important for FDG uptake.

Moreover, it is postulated that the cellular energy demand and tumor microenvironment show complex interactions, which go beyond a linear association between GLUT expression and FDG uptake alone [13].

The aim of the present analysis was to investigate the associations between GLUT 1 and GLUT 3 expression with SUV values derived from FDG-PET in a systemic review and to provide the first meta analysis of the published data.

## Materials and methods

### Data acquisition

MEDLINE and SCOPUS libraries were screened for associations between FDG-PET parameters and GLUT correlation cancer up to October 2018. The following search words were used: PET or positron emission tomography and GLUT, SUV or standardized uptake value and GLUT or glucose-transporter. Overall 292 articles were identified.

**Fig 1:**
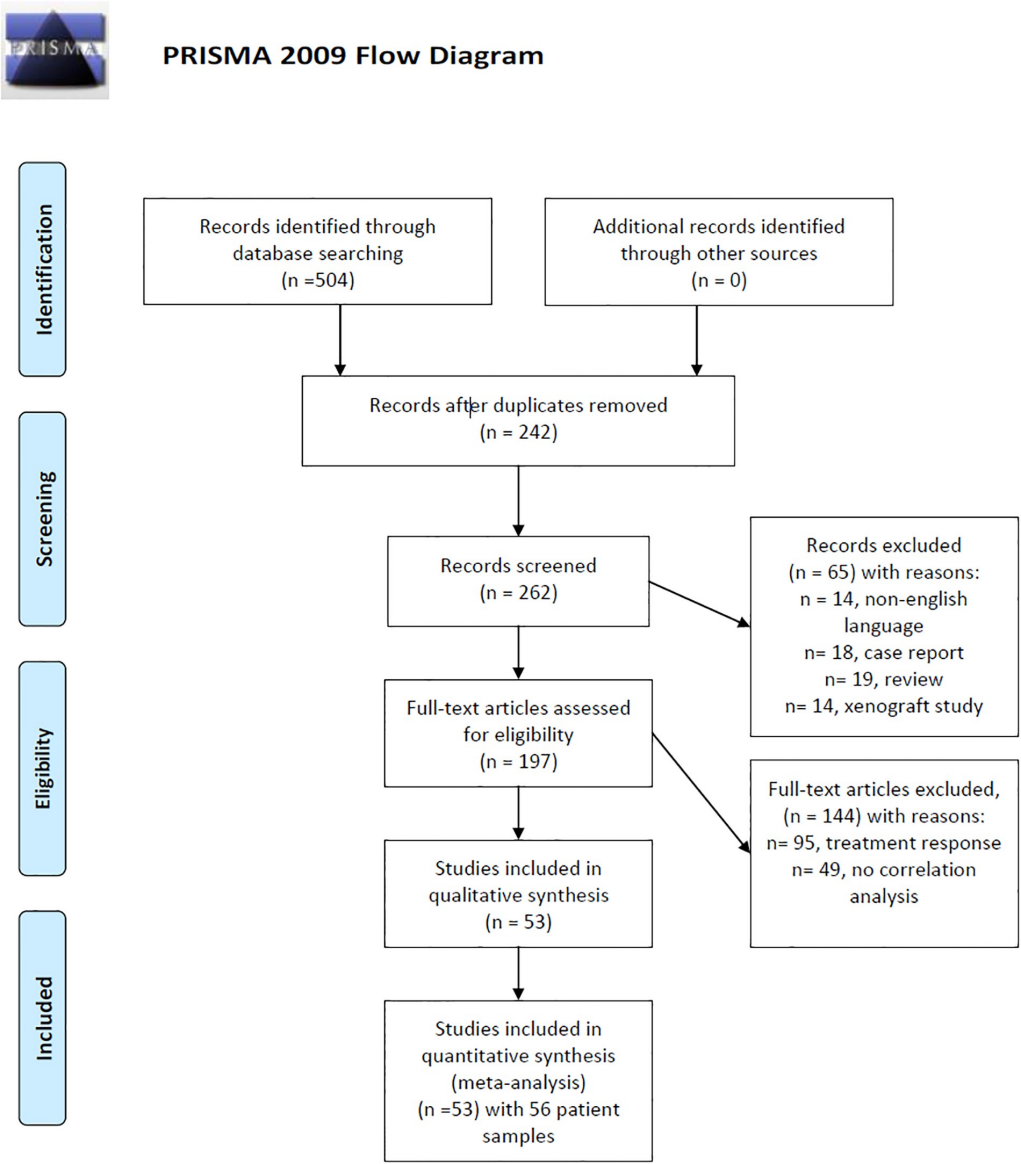
PRISMA flow chart. An overview of the paper acquisition. Finally, 53 articles were suitable for the analysis. *From*: Moher D, Liberati A, Tetzlaff J, Altman DG, The PRISMA Group (2009). *P*referred *R*eporting *I*tems for *S*ystematic *R*eviews and *M*eta-*A*nalyses: The PRISMA Statement. PLoS Med 6(7): e1000097. doi:10.1371/joumal.pmed1000097. **For more information**, **visit** www.prisma-statement.org.

After thorough review and exclusion due to doublets, review articles, case reports, non-English publications, and articles, which not contain correlation coefficients between PET and GLUT, 53 articles were suitable for the meta analysis [19–69]. In these articles 56 patient samples were acquired. Fig 1 displays the PRISMA flow chart of the paper acquisition.

The primary endpoint of the systematic review was the correlation between GLUT-1 and GLUT-3 expression with SUV values derived from FDG-PET.

Studies (or subsets of studies) were included if they satisfied all of the following criteria: (1) patients with tumor with histopathological confirmation and expression analysis of GLUT-1 and/or GLUT-3; (2) FDG-PET quantified by SUV values; (3) correlation analysis between SUV values and GLUT 1 and/or GLUT 3 expression.

Exclusion criteria were (1) systematic review (2) case reports (3) treatment prediction or histopathology performed after treatment (4) non-English language (5) xenograft or mouse/rabbit model studies.

The following data were extracted from the literature: authors, year of publication, study design, tumor entity, GLUT subtype, number of patients, and correlation coefficients.

The Preferred Reporting Items for Systematic Reviews and Meta-Analyses statement (PRISMA) was used for the paper acquisition [70].

**Fig 2:**
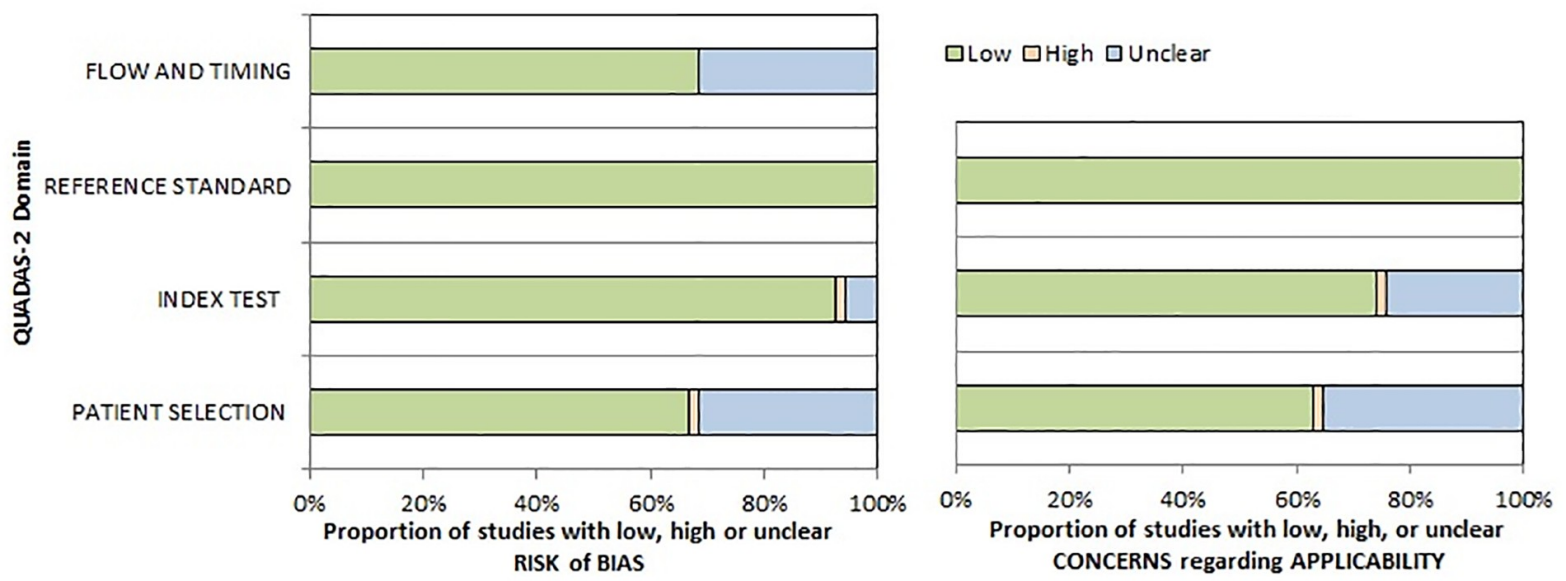
QUADAS-2 quality assessment of the included studies. There were no possible concerns of the references standard. A small amount of studies showed unclear bias regarding flow and timing, patient selection and index test.

The methodological quality of the acquired studies was independently checked by two observers (HJM and AS) using the Quality Assessment of Diagnostic Studies (QUADAS 2) instrument according to previous descriptions (Fig 2) [71].

The assessment revealed that a small portion of studies shows an unclear risk of patient selection due to non and or unclear inclusion criteria. Regarding flow and timing, some studies did not indicate whether the histopathology analysis was in a short amount of time after the PET to assure congruent results.

Associations between PET and GLUT expression were analyzed by Spearman’s correlation coefficient. The Pearson’s correlation coefficients in some studies were converted into Spearman’s correlation coefficients, as reported previously [72].

Finally, the meta-analysis was undertaken by using RevMan 5.3 (Computer Program, version 5.3, The Cochrane Collaboration, 2014, The Nordic Cochrane Centre, Copenhagen). Heterogeneity was calculated by means of the inconsistency index I^2^ [73, 74]. Additionally, DerSimonian and Laird random-effects models with inverse-variance weights were used without any further correction [75].

## Results

### Associations between SUV and GLUT 1

Overall 53 studies with 56 patient samples overall comprising 2291 patients were analyzed for the meta analysis between SUVmax and GLUT 1 expression.

There were 13 (24.5%) prospective and 40 (75.5%) retrospective study designs.

Table 1 displays the included tumor entities of the GLUT 1 analysis.

**Table 1:** Overview of the included tumor entities of the GLUT 1 analysis.

Tumor entity	N (%)
**Lung cancer**	591 (25.8)
**Head and neck cancer**	216 (9.4)
**Esophageal Cancer**	191 (8.3)
**Cervical cancer**	190 (8.3)
**Breast cancer**	175 (7.6)
**Pancreatic cancer**	127 (5.5)
**Lymph node metastasis**	99 (4.3)
**Papillary thyroid carcinoma**	94 (4.1)
**Hepatocellular carcinoma**	94 (4.1)
**Endometrial cancer**	72 (3.1)
**Sarcoma**	63 (2.8)
**Neuroendocrine tumor**	59 (2.6)
**Colorectal cancer**	57 (2.5)
**Mesenchymal uterine tumor**	47 (2.0)
**Thymic cancer**	44 (1.9)
**Gastrointestinal stromal tumor**	40 (1.8)
**Glioma**	33 (1.4)
**Pheochromacytoma**	27 (1.2)
**Bile duct cancer**	26 (1.1)
**Malignant melanoma**	19 (0.9)
**Ovarian cancer**	17 (0.8)
**Merkel cell carcinoma**	10 (0.5)
**Total**	2291 (100)

**Fig 3:**
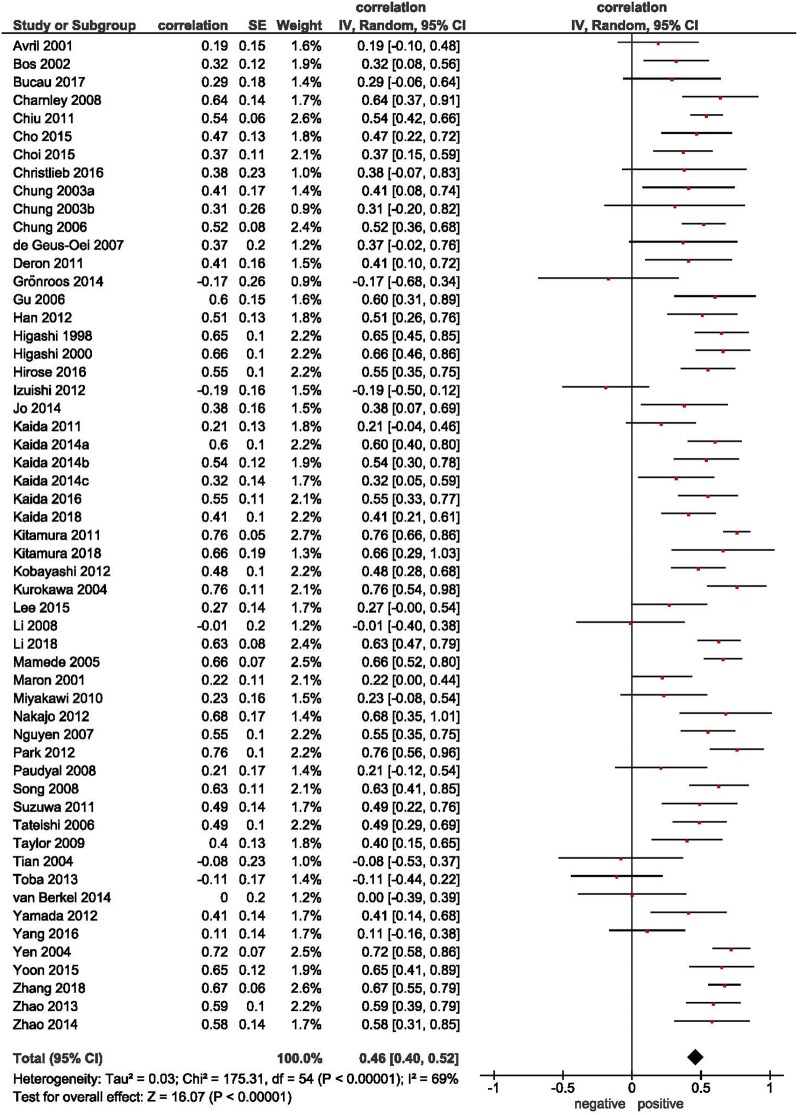
Correlation between SUVmax and GLUT 1 expression. Forrest plots of the correlations coefficients between SUVmax and GLUT 1 in all involved studies (n = 53) comprising 2291 patients. The pooled correlation coefficient was r = 0.46 (95% CI 0.40–0.52).

The overall pooled correlation coefficient of the association between SUVmax and GLUT 1 expression was r = 0.46 (95% CI 0.40–0.52) (Fig 3).

**Fig 4:**
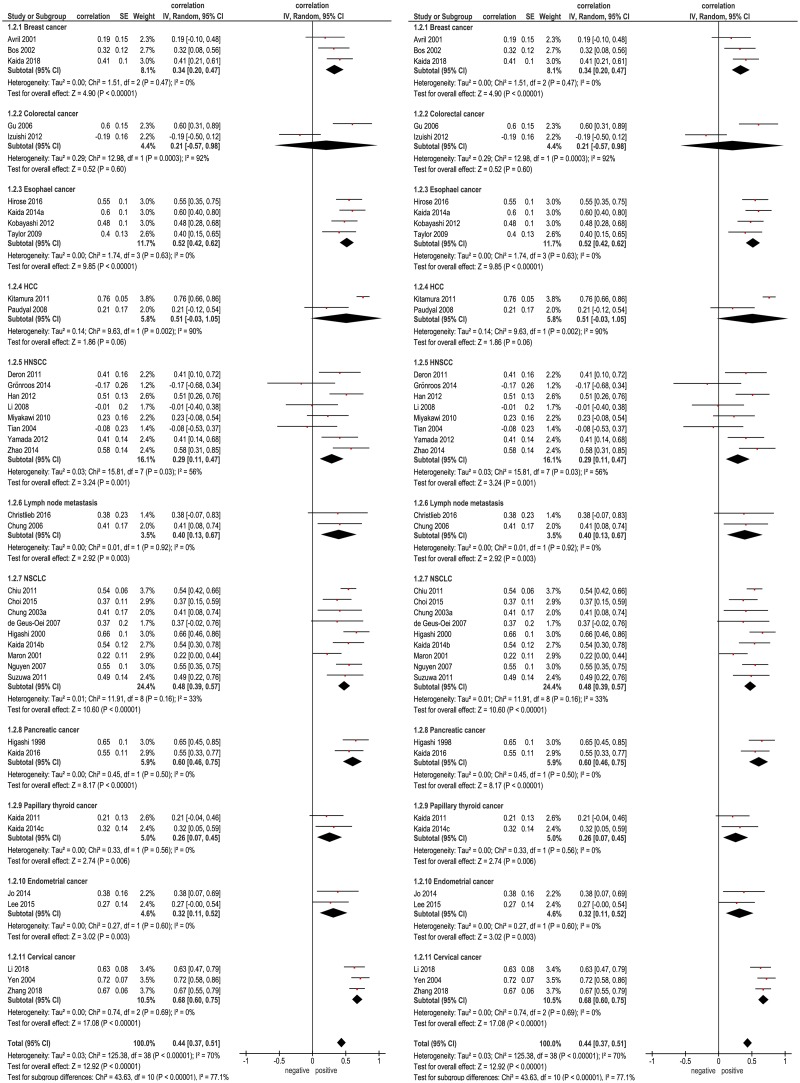
Subgroup analyses for the correlation between SUVmax and GLUT 1 expression. Forrest plots of the correlation coefficients between SUVmax and GLUT 1 in different primary tumors.

Thereafter, subgroup analyses with tumor entities comprising more than one paper were performed (Fig 4). The highest correlation coefficient was found in pancreatic cancer (r = 0.60, 95%CI 0.46–0.75), and the lowest was identified in colorectal cancer (r = 0.21 (95% CI -0.57–0.09).

### Associations between SUV and GLUT 3

**Fig 5:**
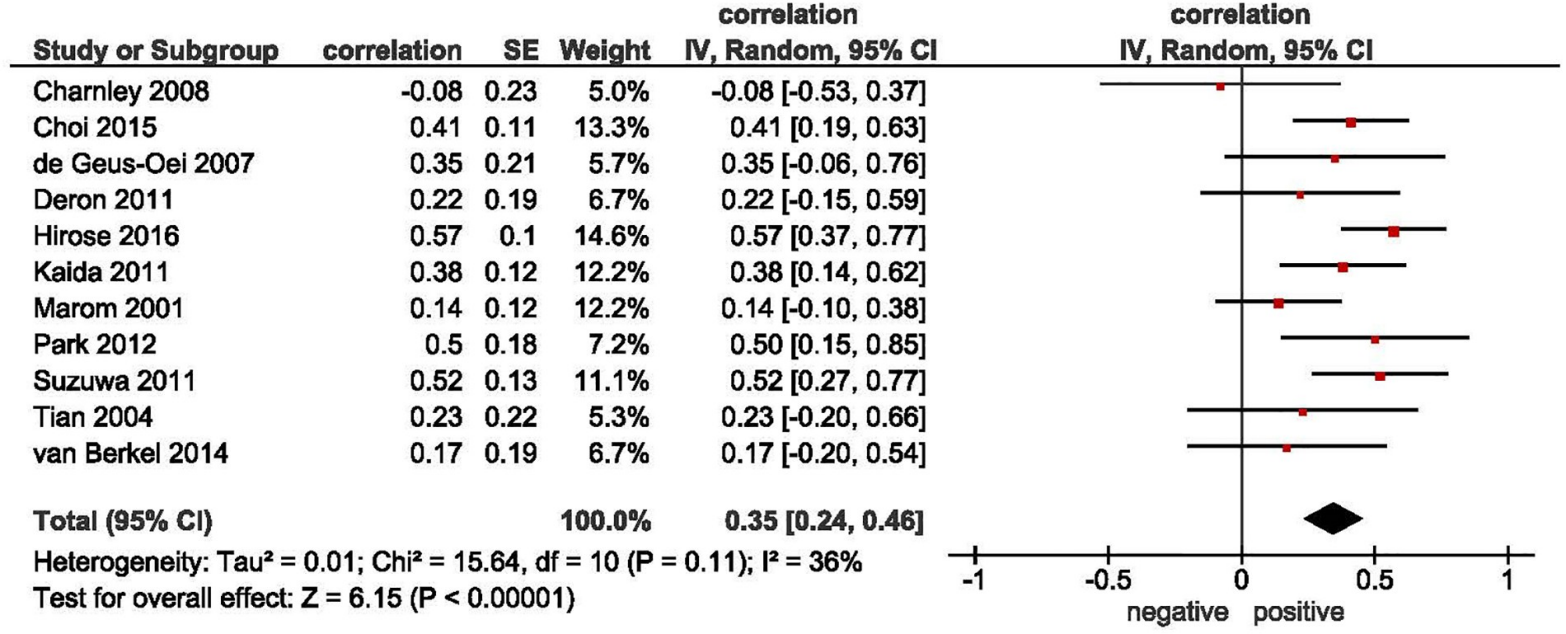
Correlation between SUVmax and GLUT 3 expression. Forrest plots of the correlations coefficients between SUVmax and GLUT 3 in 11 studies comprising 405 patients. The pooled correlation coefficient was r = 0.35 (95%CI 0.24–0.46).

**Fig 6:**
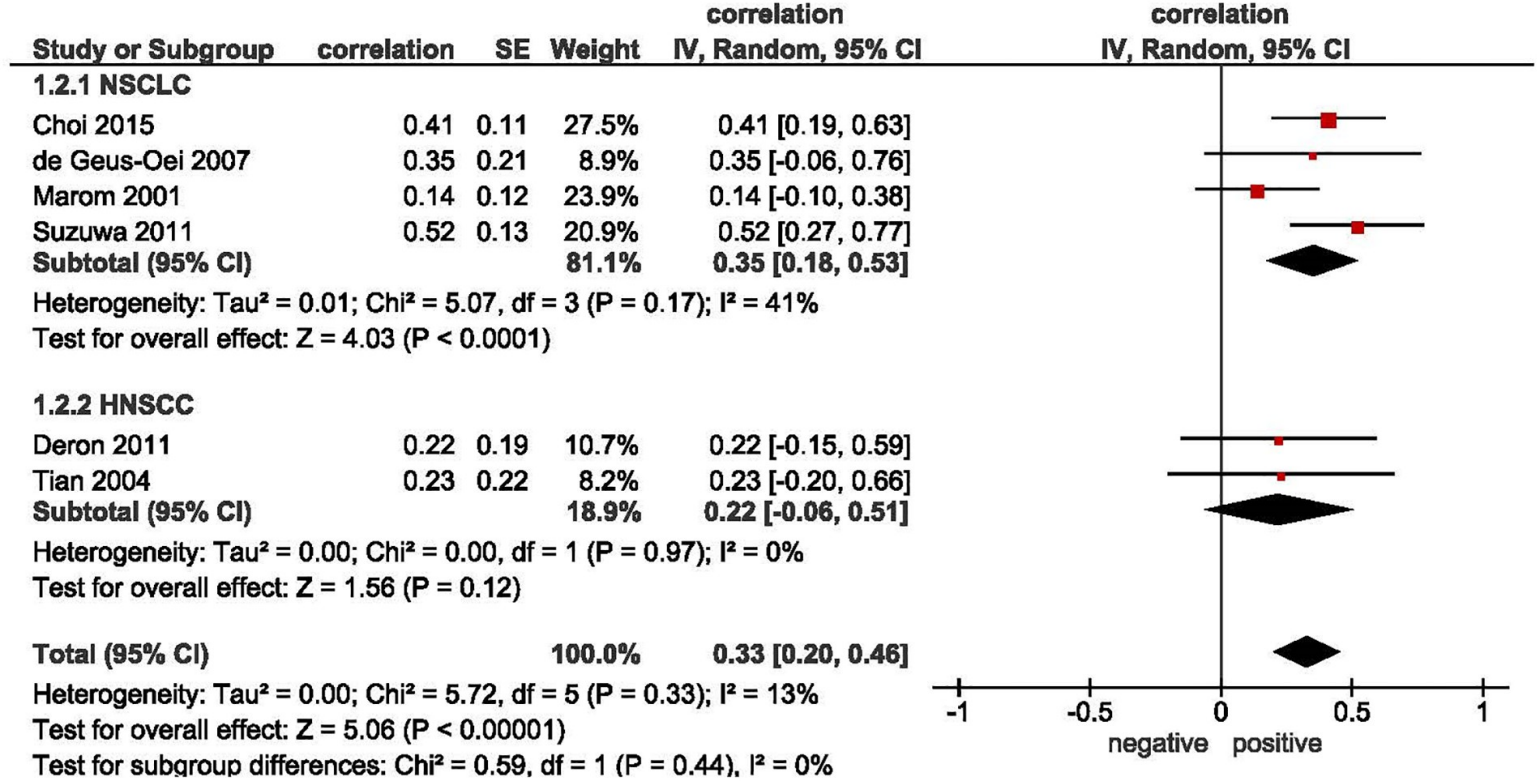
Subgroup analyses for the correlation between SUVmax and GLUT 3 expression. Forrest plots of the correlation coefficients between SUVmax and GLUT 3 in HNSCC and NSCCL subgroups.

Overall 11 studies comprising 405 patients analyzed associations between SUVmax and GLUT 3 were included into the meta analysis (Fig 5). Table 2 displays the included tumor entities. The pooled correlation coefficient was r = 0.35 (95%CI 0.24–0.46). Only 2 subgroup analyses could be performed: in non-small cell lung carcinoma (NSCLC), the correlation coefficient was r = 0.35 (95%CI 0.18–0.53) and in head and neck squamous cell carcinoma (HNSCC), it was r = 0.22 (95% CI -0.06–0.51) (Fig 6).

**Table 2:** Overview of the included tumor entities of the GLUT 3 analysis.

Tumor entity	N (%)
**Non-small cell lung cancer**	188 (46.4)
**Papillary thyroid carcinoma**	54 (13.3)
**Esophageal Cancer**	51 (12.6)
**Head and neck cancer**	46 (11.4)
**Pheochromacytoma**	27 (6.7)
**Glioma**	20 (4.9)
**Malignant melanoma**	19 (4.7)
**Total**	405 (100)

## Discussion

The present systematic review represents a meta analysis elucidating the associations between SUVmax derived from FDG-PET and GLUT expression in various tumors.

As first reported by Otto Warburg over 90 years ago, a crucial characteristic of tumor cells is an increased glucose uptake resulting in an enhanced glycolytic metabolism [3, 4]. Therefore, various tumors show an overexpression of glucose transporters (GLUTs). There are 13 types of different GLUT proteins, among which, GLUT 1 is the dominant one, which is also abundantly overexpressed in tumors [16, 76].

Previous studies analyzed possible association between SUVmax and GLUT 1 and GLUT 3. Other GLUT subtypes were only sporadically investigated and could therefore not be included into the present analysis.

Early on, it was identified that FDG uptake might be associated with GLUT expression in studies investigating lung cancer and pancreatic carcinoma [36, 37, 77]. Thus, nowadays, it is an acknowledged fact that GLUT expression is one of the main mediators of FDG uptake in tumors.

However, in the present meta analysis only a moderate association was identified between SUVmax and GLUT 1 and a weak correlation between SUVmax and GLUT 3. This fact indicates that the interactions between glucose hypermetabolism displayed by FDG-PET and glucose uptake into the cells are more complex than the sole amount of GLUT expression within the cell membranes [13]. Thus, other important proteins of the glucose metabolism, such as the hexokinase II protein might have a crucial influence on the SUV value, which has been shown in several tumor entities [78, 79]. Moreover, the FDG uptake visualized via PET might be influenced by very complex interactions of the tumor microenvironment, including inflammatory cells, extracellular matrix, microvessel density and other factors. This might be some reasons of the identified results in the present analysis.

Interestingly, the correlations between GLUT 1 and SUVmax varied significantly in different tumors. As seen, it was strong in cervical and pancreatic cancers, moderate in hepatocellular carcinoma, esophageal cancer and NSCLC, and weak in HNSCC, colorectal cancer, endometrial carcinoma and papillary thyroid cancer.

The exact cause of this phenomenon is unclear. Presumably, the above discussed complex interactions of tumor microenvironment differ between tumor types and might also influence the investigated linear association between GLUT expression and SUVmax.

For other tumor entities, such as gastric cancer, renal cell carcinoma, or urothel carcinomas, to date, there are no reports regarding associations between SUVmax and GLUT 1 or 3.

According to the literature, GLUT expression is not only specific for tumor cells. So GLUT 1 is also expressed on erythrocytes and immune cells, which induces FDG uptake also in benign diseases, for example such as lung fibrosis [14] and lung inflammatory diseases [80]. However, the inflammatory tissues might express more less GLUT 1 and consecutively display a lower SUV value than malignant tissues [81].

Moreover, a small amount of tumors might express less GLUT proteins and are, therefore, negative on PET studies, which is a very important reason for false negativity of FDG-PET. For example, this was shown in lymph node staging in lung cancer patients [82, 83]. Other reasons for PET negativity are small tumor sizes and some good differentiated tumor types [84]. Furthermore, there are some tumor entities, which are inherently known to have a low FDG uptake despite their malignant nature, such as bronchioalveolar cell carcinoma and lung carcinoids, which is believed to be causes by none or low GLUT 1 expression [85]. Consecutively, no tumors entities with such a behavior could be included into the present study.

In various studies, the important prognostic benefit of SUV values derived from PET was elucidated in several tumor entities. For example, in lung cancer patients, a higher SUVmax indicates poorer overall survival and local control as well as higher chance for distant metastases [86]. Similar results were reported for head and neck cancer [87], soft tissue sarcomas [88], and breast cancer [89]. As another aspect, SUV values can guide to evaluate treatment response, for example shown in breast cancer patients after neoadjuvant radio-chemotherapy [90].

These findings are corroborated by recent meta analyses investigating the prognostic relevance of GLUT 1 and GLUT 3 expression in tumors [91–95]. So, an overexpression of these GLUT subtypes was overall associated with a poorer prognosis in various tumors, indicated by a hazard radio of 1.63 for GLUT 1 and 1.83 for GLUT 3 [91]. This association can at last be applied to pancreatic carcinoma, gastric cancers, colorectal carcinomas, esophageal cancer, lung cancer, ovarian and uterine cancer, and oral squamous cell carcinoma [91–94]. For other tumor entities data are still lacking. Presumably, the prognostic performance of FDG-PET and GLUT expression might be linked by the associations between these parameters.

Furthermore, FDG-PET is associated with other histopathology parameters in tumors. For example, SUVmax moderately correlated with proliferation index Ki67, and might therefore be a surrogate parameter of the amount of proliferating tumor cells [96]. As another aspect, SUVmax seems to be related to vessel density in tissues, as it was exemplarily shown for lung cancer [97, 98].

Albeit the identified correlation between GLUT 1 and SUV were moderate, FDG-PET might aid in treatment response evaluation of chemotherapy targeting hypoxia-inducible factor 1 alpha, which is one of the most important mediator of metabolic gene expression including GLUT 1 [15, 16, 99–101]. Preclinical studies also elucidated the possibility of direct GLUT 1 targeting for tumor treatment, which might also be evaluated by FDG-PET. However, clinical studies are needed to proof, whether FDG-PET is capable in reflecting these treatment changes.

Moreover, FDG-PET might assess metastatic potential of tumors due to its capability to reflect the mentioned metabolic alteration, as was stated in a preclinical study [102].

There are several limitations of the present analysis to address. Firstly, most involved studies were of retrospective nature with inherent known shortcomings of this study design. Moreover, only papers published in English were included. There might be suitable papers in other languages, which were therefore not included. Secondly, different PET scanners, imaging protocols and ROI-analyses were used, which might have an influence on the correlation analysis. Thirdly, GLUT expression was estimated upon histopathology specimens, which might not be representative of the whole tumor, whereas SUVmax derived from PET represents a small area of the tumor with the highest glucose metabolism. Therefore, there might be incongruences between imaging and histopathology. Fourthly, only GLUT 1 and GLUT 3 could be included into the present analysis due to the fact that other GLUT-subtypes have not previously been investigated.

## Conclusions

In summary, the present systematic review identified only a moderate association between GLUT 1 expression and SUV values derived from FDG-PET. Moreover, the correlation between SUV and GLUT 1 varied significantly in different tumors. SUV correlated weakly with expression of GLUT 3. Presumably, the underlying mechanisms of glucose hypermetabolism in tumors are more complex and not solely depended on the GLUT expression.

## Abbreviations

FDG-PET: Fluorodeoxyglucose -Positron-emission tomography
GLUT: Glucose-transporter family
HNSCC: Head and neck squamous cell carcinoma
NSCLC: non-small-cell lung carcinoma
SUV: standardized uptake values
